# Effects of Smoothing and Adaptive Filtering in Multifocal Electroretinography (mfERG)

**DOI:** 10.3390/jcm12165286

**Published:** 2023-08-14

**Authors:** Christopher Patrick Long, Hossein Ameri

**Affiliations:** Department of Ophthalmology, USC Roski Eye Institute, Keck School of Medicine, University of Southern California, 1450 San Pablo St #4400, Los Angeles, CA 90033, USA; christopher.long@med.usc.edu

**Keywords:** multifocal electroretinography, mfERG, electrophysiologic testing, mfERG data filtering

## Abstract

(1) Background: mfERG testing is used to study the function of cone photoreceptors in the central retina. Various filters including “smoothing” (Smooth) and “adaptive data filtering” (Adapt) are used to simplify raw data. This study will seek to characterize the effect of data modification on raw patient data. (2) Methods: This was a retrospective study of patients with mfERG results at our institution. For each patient, raw mfERG data without filtering, with smooth level 4 modifier applied, and with adapt level 4 applied were collected and compared. (4) Conclusions: In all patients, smoothing and adaptive filter modifiers create statistically significant differences in both P1 latency and P1 amplitude values when compared to raw data. The impacts of these filters demonstrated in this study should impact physicians’ decision making when interpreting mfERG results.

## 1. Introduction

Electroretinography (ERG) is an ocular electrophysiologic test that allows clinicians to access the health of photoreceptors and bipolar cells in the retina [1]. Multifocal electroretinogram (mfERG) is designed specifically to examine macular photoreceptors and bipolar cells [2]. In 2021, Hoffmann et al. published the standard guidelines for the clinical use of mfERG, which continues to guide practice today [1]. 

Over the years, there have been many applications for mfERG technology [3]. As early as 1999, Maturi et al. demonstrated decreased retinal responses indicating photoreceptor damage in hydroxychloroquine toxicity, eventually becoming part of the American Academy of Ophthalmology’s official screen guidelines [4,5]. Some of the earliest uses of mfERG were in retinal dystrophies such as Stargard [6] and retinitis pigmentosa [7]. In today’s world, the clinical application of mfERG testing continues to grow and expand. In 2020, Al-Haddad et al. demonstrated the use of mfERG to help characterize the underlying pathophysiology of amblyopia [8]. In 2021, Huang et al. demonstrated the similar potential of mfERG to identify early retinal changes in diabetics without other evidence of diabetic retinopathy [9]. 

In these as well as other similar studies, analyzing mfERG results can sometimes be challenging. Considering the fine detail and precise nature of electric potentials recorded during examination, raw mfERG data can be somewhat noisy and disorganized, limiting the clinical utility of these results without any filtering. Thus, Diagnosys mfERG analysis software (Diagnosys LLC, Lowell, MA, USA) offers methods for filtering the resulting waveforms. In the software, this data filtering is available in two forms, “smoothing” and “adaptive filtering”, on a sliding scale of varying intensity from 1 to 4, with 4 being the most significant level of filtering [10]. Data “smoothing” will average an individual hexagon with the six surrounding hexagons (Diagnosys LLC, Lowell, MA, USA). Much less is known, however, about the process of “adaptive filtering”.

While other methods of cleaning raw patient testing data prior to interpretation have been evaluated, primarily spatial averaging [11], and some studies even use these filtering methods at baseline in their data analysis [12], the effects of this data filtering have yet to be fully characterized. This study seeks to characterize the effect of various degrees of data filtering applied to raw patient data obtained previously for non-research purposes as standard of care for various conditions. 

## 2. Materials and Methods

Institutional Review Board (IRB) approval was obtained for the study. The study adhered to the tenets of the Declaration of Helsinki. A retrospective chart review was performed for patients who underwent mfERG testing at our single institution. All studies were performed on the institution’s Diagnosys mfERG machine. 

All patients with mfERG testing as part of routine workup were eligible for inclusion. Patients with incomplete results were excluded. For patients eligible for inclusive, review of patient’s electronic medical record was utilized in order to record whether mfERG results were characterized as “normal” or “abnormal” clinically. 

For each patient, mfERG data were processed and extracted for analysis. The data included amplitude and implicit time of P1 or each hexagon. First, raw mfERG data without any filtering applied were collected. Next, data smoothing level 4 modifier was turned on, and the data were again extracted for analysis. Last, data smoothing level 4 was removed and, instead, adaptative filter level 4 was applied. The data were again extracted for comparison in a similar fashion. 

Data for P1 latency and P1 amplitude were analyzed from all 61 mfERG hexagons and compared between these 3 filtering statuses. Subgroup analysis included abnormal versus normal eyes, as well as central versus peripheral mfERG rings. 

In order to perform statistical analysis, IBM SPSS Statistical Software 29 (IBM Corp. Armonk, NY, USA) was utilized. The mean, standard error, and 95% confidence interval were calculated for P1 latency and P1 amplitude for raw data, Smooth 4, and Adapt 4 groups. A one-way repeated measures ANOVA was used for simultaneous comparisons between the 3 groups. A *p* value < 0.05 was used to determine statistical significance of pairwise comparisons. 

## 3. Results

### 3.1. Participants

The mfERG data of 20 patients (40 eyes) were included in the study. Six males (30%) and fourteen females (70%) were included, with a mean age of 56.82 years. Ten patients (50%) had “normal” mfERGs, and ten patients (50%) had “abnormal” mfERGs. 

### 3.2. Overall Comparison 

**Figure 1:**
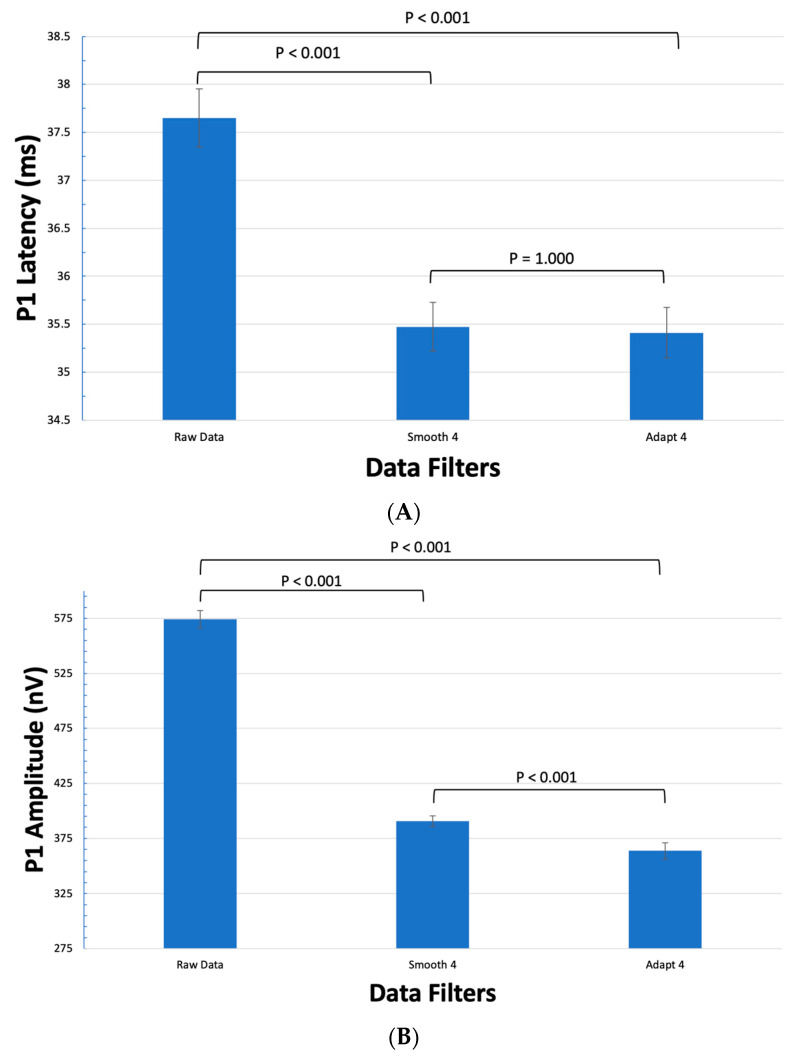
The effects of filtering on the mean latency and amplitude of all 61 hexagons. (**A**) The effects of Smooth 4 and Adapt 4 on the P1 Latency. There is a statistically significant dampening effect of both the Smooth 4 and Adapt 4 filters when compared to the raw data group. There was no statistical difference between Smooth 4 and Adapt 4 filters. (**B**) The effects of Smooth 4 and Adapt 4 on the P1 Amplitude. There is a statistically significant dampening effect of both the Smooth 4 and Adapt 4 filters when compared to the raw data group. There was also a statistically significant further dampening effect by the Adapt 4 filter when compared to the Smooth 4 filter.

When compared as a whole, Smooth 4 decreased P1 latency by 2.18 ± 0.27 ms (5.8% ± 0.7%, *p* < 0.001) while Adapt 4 decreased P1 latency by 2.39 ± 0.26 ms (5.9% ± 0.7%, *p* < 0.001) (Figure 1). Smooth 4 decreased P1 amplitude by 183.54 ± 4.92 nV (32.0% ± 0.9%, *p* < 0.001) while Adapt 4 decreased P1 amplitude by 210.59 ± 3.60 nV (36.7% ± 0.6%, *p* < 0.001) (Figure 1). While there was no significant difference between the P1 latency in Smooth 4 and Adapt 4 groups (*p* = 1.00), Adapt 4 decreased P1 amplitude by an additional 27.05 ± 4.80 nV (6.9% ± 1.2%, *p* < 0.001) when compared to Smooth 4. 

### 3.3. Subgroup Analysis

#### 3.3.1. Peripheral mfERG Ring

Subgroup analysis of the four peripheral mfERG rings is summarized in Table 1. There were significant decreases in P1 latency in both the Smooth 4 (−1.63 ± 0.40 ms, *p* < 0.001) and Adapt 4 (−2.21 ± 0.42 ms, *p* < 0.001) groups. There were also significant decreases in P1 amplitude in both Smooth 4 (−160.82 ± 7.18 nV, *p* < 0.001) and Adapt 4 (−211.89 ± 5.47 nV, *p* < 0.001) groups. While there was no significant difference between the P1 latency in Smooth 4 and Adapt 4 groups (*p* = 0.47), Adapt 4 decreased P1 amplitude by an additional 51.05 ± 7.01 nV (*p* < 0.001) when compared to Smooth 4.

#### 3.3.2. Central mfERG Rings

Subgroup analysis of the two central mfERG rings is summarized in Table 1. There were significant decreases in P1 latency in the Smooth 4 (−2.43 ± 0.80 ms, *p* = 0.01), and there was no significant difference between the raw data and Adapt 4 data (*p* = 0.14). There were significant decreases in P1 amplitude in both Smooth 4 (−202.78 ± 14.75 nV, *p* < 0.001) and Adapt 4 (−199.36 ± 10.04 nV, *p* < 0.001) groups. There was no significant difference between the P1 latency or P1 amplitude when comparing Smooth 4 and Adapt 4 groups in the central mfERG rings (*p* = 0.91, 1.00). 

#### 3.3.3. Normal Studies

Subgroup analysis of the all normal studies is summarized in Table 1. There were significant decreases in P1 latency in both the Smooth 4 (−3.05 ± 0.34 ms, *p* < 0.001) and Adapt 4 (−2.52 ± 0.33 ms, *p* < 0.001) groups. There were also significant decreases in P1 amplitude in both Smooth 4 (−115.11 ± 3.77 nV, *p* < 0.001) and Adapt 4 (−204.819 ± 3.81 nV, *p* = 0.000) groups. While there was no significant difference between the P1 latency in Smooth 4 and Adapt 4 groups (*p* = 0.17), Adapt 4 decreased P1 amplitude by an additional 89.07 ± 3.19 nV (*p* < 0.001) when compared to Smooth 4.

#### 3.3.4. Abnormal Studies 

Subgroup analysis of the all abnormal studies is summarized in Table 1. There were significant decreases in P1 latency in both the Smooth 4 (−1.31 ± 0.42 ms, *p* = 0.01) and Adapt 4 (−1.96 ± 0.40 ms, *p* < 0.001) groups. There were also significant decreased in P1 amplitude in both Smooth 4 (−251.97 ± 8.65 nV, *p* < 0.001) and Adapt 4 (−217.00 ± 6.10 nV, *p* < 0.001) groups. While there was no significant difference between the P1 latency in Smooth 4 and Adapt 4 groups (*p* = 0.42), Smooth 4 decreased P1 amplitude by an additional 34.97 ± 8.70 nV (*p* < 0.001) when compared to Adapt 4.

## 4. Discussion

Today, mfERG is widely used in our field, with applications in retinal disease ranging from retinal dystrophies to diabetic retinopathy [3,6,7,9]. For many years, researchers have studied various methods of filtering raw mfERG data, including spatial averaging, data smoothing, and pre-recording data modifiers [11,12,13]. Working towards a similar goal, Diagnosys mfERG analysis software V6.64 (Diagnosys LLC, Lowell, MA, USA) has developed a proprietary method for filtering raw patient waveforms offering user the option to apply “smoothing” and “adaptive” filters. Although these filters are widespread in use and have an impact on patient data that is obvious to the eye, quantifying the impact of these filters is an ongoing area of study. 

In this study, our results suggest these filters, when applied at extremes, are having a statistically significant impact on the result. Mainly, these filters are demonstrated to decrease P1 latency and P1 amplitude. Based on subgroup analysis, these trends are consistent in both “normal” and “abnormal” studies. This significant dampening effect also appears to be consistent in the two central mfERG rings and four peripheral mfERG rings. 

**Figure 2:**
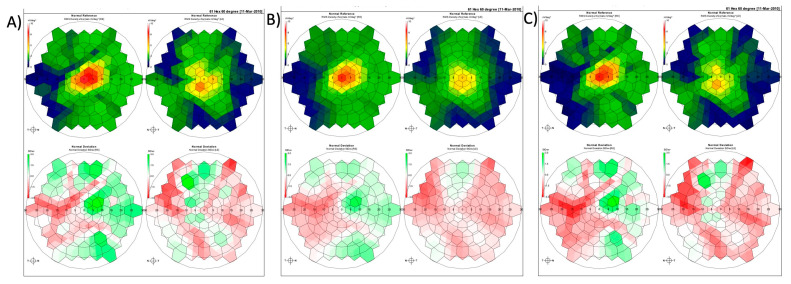
Representative normal reference (**top**) and normal deviation plots (**bottom**) from a single patient demonstrating the effects of filtering on these plots: (**A**) no filter, (**B**) smooth level 4, and (**C**) adaptive filtering level 4 on the patients right and left eye respectively.

This study is the first study to characterize the impact of these filters on raw mfERG data. However, further research is needed to identify the clinical impact of this dampening effect. Diagnosys mfERG analysis software (Diagnosys LLC, Lowell, MA, USA) produces two summary plots, normal reference and normal deviation, used as the clinical standard when interpreting mfERG results. As an example, Figure 2 shows the effects of filters on P1 latency, and amplitude on these plots translates to changes in a patient. Although the impact of these filters on the results is clear, further research is needed to characterize the effects. It is possible that these filters may impact clinical interpretation of mfERG results, causing more “normal” or “abnormal” results to appear to clinicians than the raw patient data would suggest. 

In practice today, it is likely that various institutions, as well as various research studies, are each using different data filters in their analyses of mfERG results. As demonstrated here, this may lead to variations in the study interpretations and limit the application of certain study results based on the use of different data filter settings. This study is the first to establish the dampening effect of these data filters used commonly in practice today, something physicians must consider when setting study parameters as well as interpreting mfERG results. Further work is needed to establish the full impact of these filters on the raw data, with a specific emphasis on how these filters are changing clinical interpretation of “normal” and “abnormal” studies. However, once characterized, we anticipate future research should seek to establish standard data filter settings, perhaps even customized to specific clinical and research needs. 

## Figures and Tables

**Table 1:** Subgroup analysis of P1 latency and amplitude changes in peripheral and central rings, as well as in normal and abnormal reported mfERGs.

Measure: Peripheral P1 Latency						
Setting 1	Setting 2	Mean Difference	Std. Error	*p*-value	95% Confidence Interval	
					Lower Bound	Upper Bound
Raw	Smooth 4	1.629	0.397	<0.001	0.678	2.58
Raw	Adapt 4	2.209	0.42	<0.001	1.201	3.217
Smooth 4	Adapt 4	0.58	0.408	0.47	−0.399	1.56
Measure: Central 2 Rings P1 Latency						
Setting 1	Setting 2	Mean Difference	Std. Error	*p*-value	95% Confidence Interval	
					Lower Bound	Upper Bound
Raw	Smooth 4	2.429	0.799	0.01	0.503	4.354
Raw	Adapt 4	1.683	0.842	0.14	−0.345	3.711
Smooth 4	Adapt 4	−0.745	0.724	0.91	−2.49	0.999
Measure: Peripheral P1 Amplitude						
Setting 1	Setting 2	Mean Difference	Std. Error	*p*-value	95% Confidence Interval	
					Lower Bound	Upper Bound
Raw	Smooth 4	160.836	7.179	<0.001	143.621	178.052
Raw	Adapt 4	211.890	5.472	<0.001	198.767	225.012
Smooth 4	Adapt 4	51.053	7.008	<0.001	34.246	67.86
Measure: Central 2 Rings P1 Amplitude						
Setting 1	Setting 2	Mean Difference (I-J)	Std. Error	*p*-value	95% Confidence Interval for Difference	
					Lower Bound	Upper Bound
Raw	Smooth 4	202.779	14.748	<0.001	167.259	238.298
Raw	Adapt 4	199.364	10.039	<0.001	175.184	223.544
Smooth 4	Adapt 4	−3.414	14.822	1	−39.113	32.284
Measure: P1 Latency, Normal Studies						
Setting 1	Setting 2	Mean Difference	Std. Error	*p*-value	95% Confidence Interval	
					Lower Bound	Upper Bound
Raw	Smooth 4	3.046	0.337	<0.001	2.237	3.854
Raw	Adapt 4	2.518	0.334	<0.001	1.718	3.318
Smooth 4	Adapt 4	−0.527	0.277	0.17	−1.191	0.136
Measure: P1 Latency, Abnormal Studies						
Setting 1	Setting 2	Mean Difference	Std. Error	*p*-value	95% Confidence Interval	
					Lower Bound	Upper Bound
Raw	Smooth 4	1.306	0.419	0.01	0.301	2.312
Raw	Adapt 4	1.958	0.404	<0.001	0.989	2.927
Smooth 4	Adapt 4	0.651	0.439	0.42	−0.402	1.705
Measure: P1 Amplitude, Normal Studies						
Setting 1	Setting 2	Mean Difference	Std. Error	*p*-value	95% Confidence Interval	
					Lower Bound	Upper Bound
Raw	Smooth 4	115.111	3.769	<0.001	106.075	124.148
Raw	Adapt 4	204.188	3.812	0	195.049	213.326
Smooth 4	Adapt 4	89.076	3.193	<0.001	81.422	96.73
Measure: P1 Amplitude, Abnormal Studies						
Setting 1	Setting 2	Mean Difference	Std. Error	*p*-value	95% Confidence Interval	
					Lower Bound	Upper Bound
Raw	Smooth 4	251.970	8.652	<0.001	231.229	272.711
Raw	Adapt 4	217.000	6.1	<0.001	202.376	231.624
Smooth 4	Adapt 4	−34.970	8.697	<0.001	−55.82	−14.12

## Data Availability

Not applicable.
